# Association between frailty status and osteomyelitis: A nested case-control study

**DOI:** 10.1371/journal.pone.0350395

**Published:** 2026-06-01

**Authors:** Can Guo, Xukun Luo, Zijin Tang, Jian Zhou, Tang Liu

**Affiliations:** 1 Department of Spine Surgery, The Second Xiangya Hospital of Central South University, Changsha, China; 2 Clinical Medicine Eight-year Program, 2302 Class, 2023 Grade, Central South University, Changsha, China; 3 Department of Orthopedics, The Second Xiangya Hospital of Central South University, Changsha, China; 4 National Clinical Research Center for Metabolic Diseases, The Second Xiangya Hospital of Central South University, Changsha, China; 5 Postdoctoral Mobile Station of Clinical Medicine, The Second Xiangya Hospital of Central South University, Changsha, China; Southern Medical University Nanfang Hospital, CHINA

## Abstract

**Background:**

Although frailty has been recognized as a predictor of various adverse outcomes in older adults, the association between physical pre-frailty or frailty and the risk of osteomyelitis remains unclear.

**Methods:**

In this nested case-control study, data from 466,918 eligible participants recruited into the UK Biobank between 2006 and 2010 were used. Incident osteomyelitis cases were identified through linked electronic health records up to 19 December 2022 and matched to controls at a 1:5 ratio by age, sex, and assessment center from the baseline population meeting the eligibility criteria. Frailty status was assessed at baseline using a five-item phenotype adapted from the Fried criteria and categorized as non-frail, pre-frail, or frail. Conditional logistic regression was used to evaluate the association between frailty status and osteomyelitis after adjustment for socioeconomic factors, lifestyle behaviors, medication use, and clinical risk factors.

**Results:**

Compared with non-frail individuals, participants with physical pre-frailty had a significantly higher odds of osteomyelitis (OR = 1.38; 95% CI: 1.16–1.64), with the odds further increasing among those with physical frailty (OR = 2.79; 95% CI: 2.05–3.81), demonstrating a clear dose–response relationship (P-trend <0.001). These associations between physical frailty status and osteomyelitis remained robust across subgroups defined by potential risk factors.

**Conclusions:**

Physical pre-frailty and frailty were associated with higher odds of osteomyelitis, with the odds increasing across frailty categories.

## Introduction

Osteomyelitis refers to bone deterioration resulting from bacterial infection [1]. With the growing number of older adults and patients with chronic diseases, the epidemiology of osteomyelitis has changed. A 40-year population-based study in Minnesota, USA, showed a rising incidence, especially among men aged over 65 [2,3]. Commonly affected sites include the spine, pelvis, and long bones. Major causes are trauma, diabetic foot, and surgery-related infections [4–6]. Despite progress in antimicrobial treatment, challenges remain due to resistant strains, biofilm formation, poor antibiotic penetration, and variability in host immunity [7]. These features not only complicate clinical management but also underscore the importance of identifying individuals at increased risk before severe infection develops. Early identification and intervention in high-risk groups may therefore help improve outcomes and reduce healthcare burden.

Although population-specific and disease-adapted frailty models are emerging [8], the most widely used remains the Fried phenotype, which includes five criteria: unintentional weight loss, exhaustion, low physical activity, slow walking speed, and weak grip strength. Individuals meeting three or more criteria are classified as frail, while those with one or two are considered pre-frail [9,10]. Existing studies have linked frailty to a higher risk of fractures and to postoperative infections related to implants [11,12]. Multiple chronic conditions, such as diabetes, are established risk factors for osteomyelitis, and fracture type itself is also considered an independent predictor [13]. Frailty not only predicts multimorbidity but is also closely associated with many chronic diseases [14]. However, studies on the association between frailty and osteomyelitis remain limited, and no nested cohort study has systematically evaluated this relationship.

In this study, we aimed to investigate the association between physical frailty status and osteomyelitis. We further examined the potential interaction between physical frailty and other risk factors in relation to osteomyelitis.

## Methods

### Data source

This study was conducted as a nested case-control analysis within the UK Biobank. UK Biobank is a large-scale prospective cohort study designed to investigate the mechanisms underlying complex diseases using multidimensional health data from middle-aged and older adults. About 500,000 participants aged 40–69 were recruited across the UK, including urban and rural areas. Its key strength is the multi-level integration of genomic, metabolomic, and imaging biomarkers with detailed lifestyle, psychosocial, and environmental data, together with long-term electronic health record (EHR) follow-up. Ongoing linkage to national health databases and regular updates support research on chronic and ageing-related conditions, as well as behavioural and environmental health. Its open-access policy enables interdisciplinary and global research on key public health questions [15,16].

UK Biobank data were accessed for research purposes under approved Application No. 80610. The authors did not have access to any information that could identify individual participants, and all analyses were conducted using anonymized data.

### Study design and population

**Fig 1:**
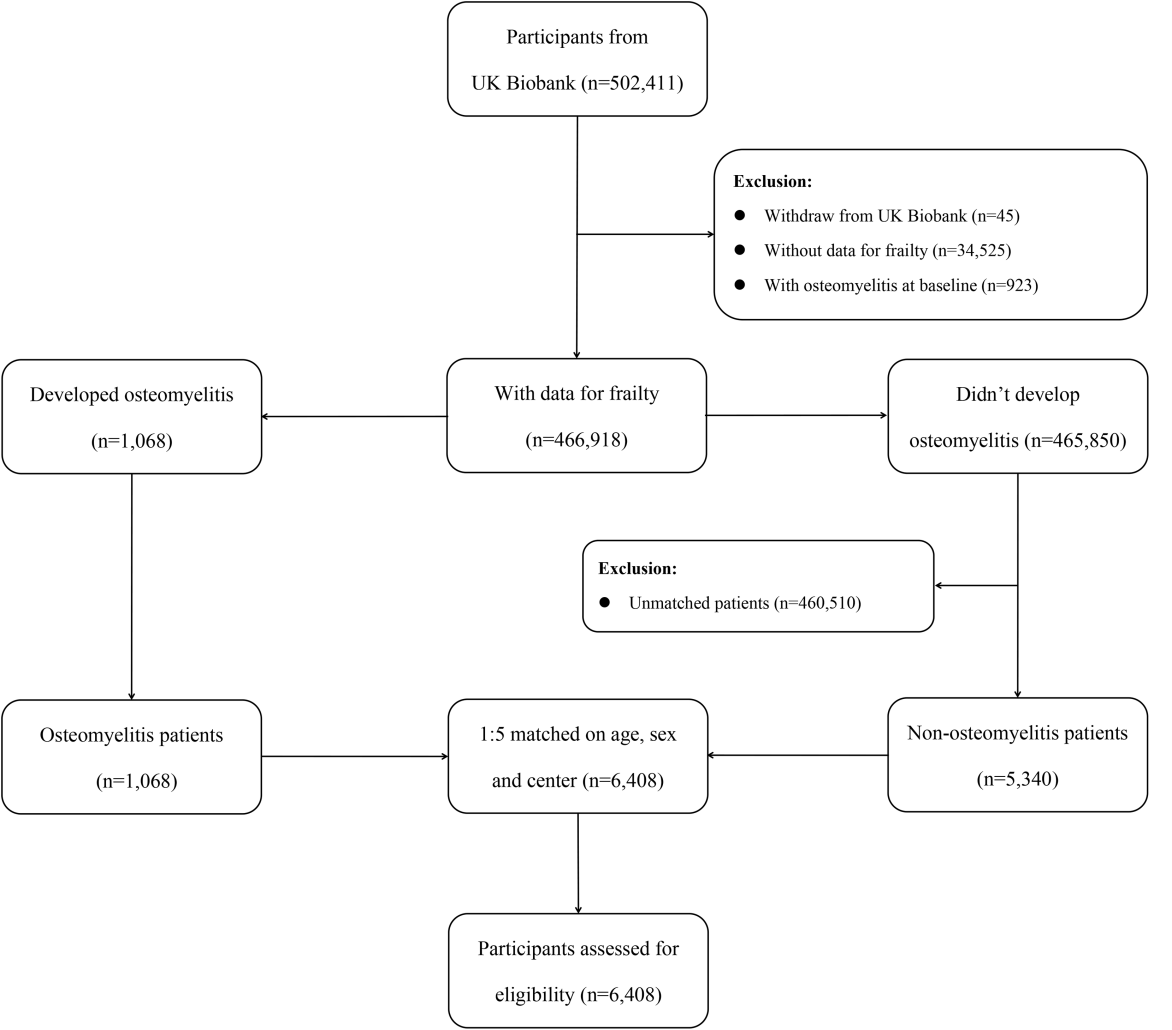
Flowchart of participant selection..

We conducted a nested case–control study within the UK Biobank cohort between baseline recruitment (between 2006 and 2010) and December 19, 2022. The study population initially included 502,411 participants. We excluded participants who had withdrawn from follow-up (n = 45), those without frailty data (n = 34,525), and those with osteomyelitis at baseline (n = 923), leaving 466,918 eligible participants. Cases were participants with incident osteomyelitis identified during follow-up. The index date was defined as the date of incident osteomyelitis. Each case was matched, using risk-set sampling, on age, sex, and assessment center with up to five controls who were alive, under follow-up, and free of osteomyelitis on the index date. This yielded a final sample of 1,068 osteomyelitis cases and 5,340 matched controls (Fig 1). All participants were followed from the baseline assessment until the first diagnosis of osteomyelitis, death, loss to follow-up, or 19 December 2022, whichever occurred first.

### Assessment of frailty

Physical frailty status was assessed based on five criteria proposed by Fried et al [9]; operational definitions and UK Biobank field IDs are provided in S1 Table. Each criterion was scored 1 if present (otherwise 0): (i) weight loss (“lost weight”); (ii) exhaustion (“more than half the days” or “nearly every day”); (iii) low physical activity (no activity or only light activity ≤ once/week); (iv) slow usual walking pace; and (v) weak handgrip strength measured with a hydraulic dynamometer, sex- and Body mass index (BMI)-adjusted (lowest quintile within sex–BMI strata). Frailty index scores (range 0–5) were summed and categorised as non-frail (0), pre-frail (1–2) and frail (3–5).

### Assessment of other variables

Covariate information was obtained from standardized touchscreen questionnaires, physical measurements, and biochemical assays collected at UK Biobank assessment centers. Variables included sociodemographic characteristics, lifestyle behaviors, nutritional supplementation, physical status, clinical risk factors, and area-level deprivation.

Covariates included age, height, sex, and ethnicity (White/non-White), which were self-reported at baseline and verified by staff. Socioeconomic status was measured using the Townsend Deprivation Index derived from residential postcodes, reflecting area-level unemployment, housing, car ownership, and overcrowding; higher scores indicate greater deprivation. Educational attainment was based on self-reported highest qualification and categorised as no formal qualifications, secondary education, or higher education. BMI was calculated as weight divided by height squared (kg/m²) from standardised measurements (Seca stadiometers/scales) and grouped as <18.5, 18.5–30, or ≥30 kg/m². Smoking status was self-reported as never, former, or current. Alcohol consumption frequency was the number of drinking occasions per week, categorised as <3 or ≥3 times/week. Healthy diet score followed a published method [15] (range 0–5): 1 point for vegetable ≥4 tbsp/day, fruit ≥3/day, fish ≥2/week, unprocessed red meat ≤2/week, and processed meat ≤2/week. Supplement use, including calcium and vitamin D, was self-reported (never/previous/current) and dichotomised (use vs non-use); calcium and vitamin D were included as binary variables (yes/no) to indicate nutritional intervention.

We further included several important clinical risk factors related to the occurrence of osteomyelitis, including diabetes mellitus, chronic kidney disease, immunosuppression, history of trauma or surgery, multimorbidity, and sickle cell disease. Multimorbidity was used to reflect the overall burden of multiple chronic conditions in an individual. History of trauma or surgery was derived from UK Biobank field n_136_0_0, a baseline self-reported variable indicating whether the participant had a prior history of trauma or surgery. The other clinical conditions were identified from linked hospital inpatient records using the International Classification of Diseases, 10th Revision (ICD-10) codes, as detailed in S2 Table.

All covariates were considered potential confounders and adjusted for in multivariable models. Detailed documentation on variable collection is available on the UK Biobank website (www.ukbiobank.ac.uk).

### Outcome Assessment

The diagnosis of osteomyelitis was identified using ICD-10 codes (S2 Table), including M86 (osteomyelitis, encompassing acute, subacute, and chronic forms) and M46.2 (osteomyelitis of vertebra). The date of osteomyelitis onset was determined from cumulative hospital inpatient records.

Furthermore, the information on reasons and dates for hospitalization can be used through the linkage to Scottish morbidity records for Scottish participants and health event statistics for England and Wales participants. More information is accessible at https://digital.nhs.uk/ser-vices.

### Statistical analysis

Continuous variables were expressed as the mean ± SD, and all categorical variables were expressed as the count with percentage. Four conditional logistic regression models were constructed to analyze the association of frailty status with osteomyelitis. Model 1 was adjusted for baseline age (years) and sex (male or female). Model 2 was further adjusted for ethnicity, Townsend Deprivation Index, educational attainment, body mass index, smoking status, alcohol frequency, and healthy diet score. Model 3 was further adjusted for vitamin D and calcium supplementation. Model 4 was further adjusted for diabetes mellitus, chronic kidney disease, immunosuppression, history of trauma or surgery, multimorbidity, and sickle cell disease. For covariates with missing data, categorical variables were assigned to an additional “missing” category, while continuous variables were imputed using mean values. The number and percentage of participants with missing covariate data are shown in S3 Table.

We performed a series of subgroup analyses stratified by age, sex, BMI, Townsend Deprivation Index, education, smoking, alcohol, diet score, diabetes mellitus, chronic kidney disease, and immunosuppression. We used the same conditional logistic regression model by adding interaction terms.

### Sensitivity analyses

We performed three sensitivity analyses to assess the robustness of the results. First, participants who reported bone fractures within the first 2 years of follow-up were excluded. Second, analyses were repeated after excluding individuals with missing covariate data. Third, missing data were additionally addressed using multiple imputation with chained equations.

All sensitivity analyses reported ORs with 95% CIs. If the estimates across different strategies were directionally consistent and of similar magnitude, the findings were considered robust. All statistical analyses were performed using SAS version 9.4 (SAS Institute, Cary, NC) and R version 4.2.0 (www.r-project.org). A 2-sided *P*-value <0.05 was considered statistically significant.

### Ethics statement

This research was conducted under UK Biobank Application Number 80610. UK Biobank has ethical approval from the North West Multi-centre Research Ethics Committee (11/NW/0382) and therefore no separate ethical approval was required.

## Results

### Baseline characteristics

Table 1 presents the baseline characteristics of the 6,408 participants included in this nested case-control study, comprising 1,068 incident osteomyelitis cases and 5,340 matched controls. All participants were drawn from the UK Biobank cohort and had available data on frailty assessment. The mean age was 59.6 years, and the mean height was 170.8 cm; 34.2% of participants were female. In the case group, 379 (35.5%) participants were non-frail, 531 (49.7%) pre-frail, and 158 (14.8%) frail. Compared with controls, cases had higher proportions of all five physical frailty components. They also had higher Townsend Deprivation Index scores, lower rates of higher education, higher BMI, more current smokers, poorer diet quality, and slightly more frequent use of vitamin D and calcium supplements. In addition, cases had higher prevalences of diabetes mellitus, chronic kidney disease, immunosuppression, history of trauma or surgery, and multimorbidity, whereas sickle cell disease was rare in both groups. No substantial differences were observed between the groups in age, height, or ethnic composition.

**Table 1:** Baseline features of participants.

Characteristics	Total (n = 6408)	Case (n = 1068)	Control (n = 5340)
Physical frailty indicators, n (%)			
Weight loss	1025 (16.0)	238 (22.3)	787 (14.7)
Exhaustion	786 (12.3)	219 (20.5)	567 (10.6)
Low physical activity	683 (10.7)	219 (20.5)	464 (8.7)
Slow walking pace	772 (12.1)	295 (27.6)	477 (8.9)
Low grip strength	1056 (16.5)	283 (26.5)	773 (14.5)
Age, years, mean (SD)	59.6 (7.43)	59.6 (7.4)	59.6 (7.4)
Height, cm, mean (SD)	170.8 (9.0)	171.2 (9.4)	170.7 (8.9)
Female, n (%)	2190 (34.2)	365 (34.2)	1825 (34.2)
White ethnic, n (%)	5873 (91.8)	985 (92.2)	4888 (91.5)
Townsend Deprivation Index, mean (SD)	−1.23 (3.20)	−0.3 (3.6)	−1.42 (3.1)
Education level, n (%)			
Low level	2687 (42.4)	506(43.3)	2181 (41.3)
Medium level	1760 (27.8)	308(43.6)	1452 (27.5)
High level	1890 (29.8)	238(13.2)	1652 (31.3)
Body mass index (kg/m^2^), n (%)			
<18.5	31 (0.5)	5 (0.5)	26 (0.5)
18.5- < 30	4618 (72.1)	613 (57.4)	4005 (75)
≥30	1759 (27.5)	450 (42.1)	1309 (24.51)
Smoking status, n (%)			
Never	4114 (48.7)	460 (43.3)	3654 (49.8)
Previous	2598 (40.7)	463 (43.6)	2135 (40.1)
Current	679 (10.6)	140 (13.2)	539 (10.1)
Alcohol intake, times/week, n (%)			
<3	3355 (52.4)	633 (59.3)	2722 (51.0)
≥3	3046 (47.6)	434 (40.7)	2612 (49.0)
Healthy diet score, n (%)			
<3	2202 (35.8)	387 (38.2)	1815 (35.3)
≥3	3949 (64.2)	625 (61.8)	3324 (64.7)
Vitamin D, n (%)	228 (3.6)	49 (4.6)	179 (3.4)
Calcium supplementation, n (%)	381 (6.0)	69 (6.5)	312 (5.9)
Diabetes mellitus, n (%)	1130(17.63)	498(46.63)	632(11.84)
Chronic kidney disease, n (%)	708(11.04)	322(30.15)	386(7.23)
Immunosuppression, n (%)	1684(26.28)	573(53.65)	1111(20.81)
History of trauma or surgery, n (%)	5127(80.01)	927(86.8)	4200(78.65)
Multimorbidity, n (%)	4186(65.32)	958(89.7)	3228(60.45)
Sickle cell disease, n (%)	3(0.05)	1(0.09)	2(0.04)

n, number of participants; SD, standard deviation.

### The association between osteomyelitis and frailty

The median follow-up time to incident osteomyelitis was 13.5 years. Conditional logistic regression model was used to assess the association between frailty status and osteomyelitis (Table 2). Among participants with non-frailty, pre-frailty, and frailty, the numbers of cases (controls) were 379 (3,142), 531 (2,029), and 158 (169), respectively (P-trend < 0.001). In Model 1, adjusted for age and sex, we observed a significant association between physical pre-frailty and frailty with osteomyelitis. The OR was 2.23 (1.93–2.57) for pre-frailty and 7.98 (6.23–10.23) for frailty, respectively (P-trend < 0.001). After further adjusted for ethnicity, Townsend Deprivation Index, education, BMI, smoking status, alcohol consumption frequency, healthy diet score, vitamin D, calcium supplementation, diabetes mellitus, chronic kidney disease, immunosuppression, history of trauma or surgery, multimorbidity, and sickle cell disease, the OR estimates remained largely unchanged.

**Table 2:** Association of physical frailty status with osteomyelitis.

	Case/Control	OR (95% CI)			
		Basic model 1^a^	Multivariable model 2^b^	Multivariable model 3^c^	Multivariable model 4^d^
Non-frailty	379/3142	1.00 (Reference)	1.00 (Reference)	1.00 (Reference)	1.00 (Reference)
Pre-frailty	531/2029	2.23 (1.93-2.57) ^*^	1.92 (1.64-2.23) ^*^	1.91 (1.64-2.23) ^*^	1.38 (1.16-1.64) ^*^
Frailty	158/169	7.98 (6.23-10.23) ^*^	5.85 (4.47-7.65) ^*^	5.77 (4.40-7.55) ^*^	2.79 (2.05-3.81) ^*^
*P*-trend		<0.001	<0.001	<0.001	<0.001

^a^Adjusted for age (years) and sex (male or female).

^b^Basic model + ethnic background, Townsend Deprivation Index, education level, body mass index, smoking status, alcohol intake, healthy diet score.

^c^Model 2 + vitamin D supplementation, calcium supplementation.

^d^Model 3 + diabetes mellitus, chronic kidney disease, immunosuppression, history of trauma or surgery, multimorbidity, and sickle cell disease

* *P*-value < 0.001

CI, confidence interval; OR, odds ratio.

**Fig 2:**
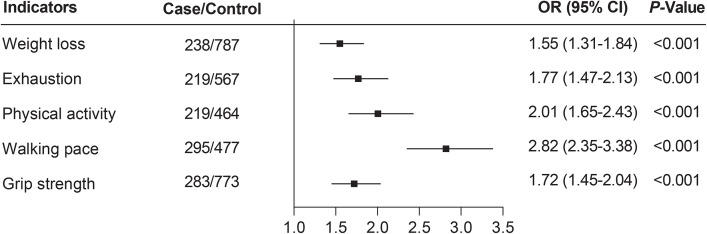
Odds ratios (ORs) for the associations between the five physical frailty components and osteomyelitis, derived from Model 4 was adjusted for age, sex, ethnicity, socioeconomic status, educational attainment, body mass index, smoking status, alcohol intake, healthy diet score, vitamin D supplementation, calcium supplementation, diabetes mellitus, chronic kidney disease, immunosuppression, history of trauma or surgery, multimorbidity, and sickle cell disease.

We also evaluated the associations between the five individual frailty components and the risk of osteomyelitis in Model 4. As shown in Fig 2, all components were significantly associated with increased osteomyelitis risk (all P < 0.001), with slow walking pace showing the strongest association (OR = 2.82, 95% CI: 2.35–3.38).

### Stratified analyses

We performed stratified analyses of the association between frailty status and osteomyelitis according to multiple potential risk factors, including age, sex, Townsend Deprivation Index, height, body mass index, smoking status, alcohol intake, dietary quality, diabetes mellitus, chronic kidney disease, immunosuppression, history of trauma or surgery, and multimorbidity. Stratified analyses showed that the association was generally consistent across most subpopulations. No statistically significant interactions were observed for most factors (Table 3); However, the results from stratified analyses demonstrated that the association of physical pre-frailty and frailty with osteomyelitis was strongest among participants aged <60 years (P for interaction = 0.02).

**Table 3:** Association of physical frailty status with osteomyelitis stratified by potential risk factors via model 4^*^.

Subgroup	Physical frailty status			*P*-trend	*P-*interaction
	Non-frailty	Pre-frailty	Frailty		
Age (years)					0.02
<60	1 (references)	1.68 (1.27-2.25)	3.69 (2.18-6.25)	<0.001	
≥60	1 (references)	1.24 (0.99-1.54)	2.42 (1.63-3.59)	<0.001	
Sex					0.77
Women	1 (references)	1.44 (1.07-1.93)	2.59 (1.52-4.39)	<0.001	
Men	1 (references)	1. 34(1.09-1.66)	3.03 (2.05-4.48)	<0.001	
Townsend deprivation index					0.72
<Median	1 (references)	1.36 (0.99-1.86)	2.94 (1.43-6.06)	<0.001	
≥Median	1 (references)	1.26 (0.97-1.65)	2.74 (1.65-4.28)	<0.001	
Height (cm)					0.61
<170	1 (references)	1.38 (0.99-1.93)	2.56 (1.51-4.35)		
≥170	1 (references)	1.47 (1.14-1.91)	4.13 (2.19-7.80)		
Body mass index (kg/m^2^)					0.19
<30	1 (references)	1.66 (1.32-2.10)	4.29 (2.46-7.50)	<0.001	
≥30	1 (references)	0.91 (0.61-1.36)	1.96 (1.05-3.66)	<0.001	
Education (years)					0.45
<15	1 (references)	1.45 (1.05-1.95)	3.26 (1.88-5.64)		
≥15	1 (references)	1.55 (1.17-2.05)	2.63 (1.34-5.16)		
Smoking status					0.40
Never	1 (references)	1.20 (0.86-1.66)	1.73 (0.92-3.27)	<0.001	
Ever	1 (references)	1.41 (1.08-1.84)	3.96 (2.41-6.52)	<0.001	
Alcohol intake (times/week)					0.55
<3	1 (references)	1.56 (1.20-2.04)	2.73 (1.74-4.28)	<0.001	
≥3	1 (references)	1.51 (1.10-2.06)	3.55 (1.67-7.48)	<0.001	
Healthy diet score					0.15
<3	1 (references)	2.24 (1.46-3.44)	5.61 (2.61-12.06)	<0.001	
≥3	1 (references)	1.15(0.90-1.48)	2.69 (1.58-4.58)	<0.001	
Diabetes mellitus					0.06
No	1 (references)	1.53 (1.23-1.89)	3.11 (1.96-4.93)	<0.001	
Yes	1 (references)	1.04 (0.64-1.70)	2.50 (1.21-5.16)	<0.001	
Chronic kidney disease					0.83
No	1 (references)	1.33 (1.09-1.61)	2.61 (1.78-3.85)	<0.001	
Yes	1 (references)	1.47 (0.64-3.37)	4.18 (1.24-14.04)	<0.001	
Immunosuppression					0.23
No	1 (references)	1.53 (1.20-1.96)	3.86 (2.27-6.55)	<0.001	
Yes	1 (references)	0.90 (0.61-1.32)	2.10 (1.15-3.80)	<0.001	

* Model 4: adjusted for age (years), sex (male or female), ethnic background, Townsend Deprivation Index, education level, body mass index, smoking status, alcohol intake, healthy diet score, vitamin D supplementation, calcium supplementation, diabetes mellitus, chronic kidney disease, immunosuppression, history of trauma or surgery, multimorbidity, and sickle cell disease.

### Sensitivity analyses

To evaluate the robustness of our findings, several sensitivity analyses were performed. The results remained stable after excluding participants who developed osteomyelitis within the first two years of follow-up (S4 Table). Furthermore, excluding individuals with missing covariate data did not materially alter the findings (S5 Table). Consistent results were also observed after performing multiple imputation for missing covariates (S6 Table).

## Discussion

In this nested case-control study including 1,068 cases and 5,340 controls, we observed significant associations of physical pre-frailty and frailty with osteomyelitis. Compared with non-frail individuals, those classified as pre-frail and frail had 1.37-fold and 2.79-fold higher odds of osteomyelitis, respectively. More importantly, these associations remained robust after adjustment for a wide range of potential confounders. These findings suggest that frailty, as a measurable clinical condition, may help identify individuals at higher risk of osteomyelitis. Further analyses revealed that among the individual frailty components, “slow walking pace” and “low physical activity” showed relatively stronger associations with osteomyelitis, suggesting that impaired physical function may be an important factor underlying the association between frailty and osteomyelitis. This finding is broadly consistent with previous observations in bone-related infection research. Previous studies have shown that limited mobility, a greater burden of chronic disease, and reduced physiological reserve are associated with a higher risk of bone infection, delayed healing, and more severe functional impairment. In older adults, these unfavorable factors may also contribute to a higher susceptibility to vertebral osteomyelitis [17]. From a physiological perspective, physical activity may promote osteocyte activity and improve local blood supply through mechanical stimulation, thereby helping to maintain skeletal metabolic homeostasis and local defense capacity. In contrast, chronic osteomyelitis often develops in environments characterized by structural bone destruction, impaired perfusion, and local hypoxia, and these adverse conditions may be further aggravated by reduced physical activity [7]. In addition, low physical activity may be associated not only with a higher risk of infection onset but also with poorer post-infection outcomes. A multicenter cohort study from Asia showed that patients with poor postoperative ambulation had a higher recurrence rate of osteomyelitis, suggesting that functional status may be involved in both the onset and subsequent progression of infection [18]. At the same time, functional decline is often accompanied by reduced muscle mass, weakened immune responsiveness, and lower overall physiological tolerance, all of which may further compromise the host response to infection. Previous studies have also suggested that individuals with marked functional impairment are more likely to develop severe infections such as osteomyelitis and to experience a more prolonged disease course or worse clinical outcomes [19,20]. Taken together, our findings are consistent the possibility that the reduced physical function and diminished physiological reserve reflected by frailty may be relevant to the observed higher susceptibility to osteomyelitis.

In addition to the associations observed with individual frailty components, the overall frailty status demonstrated a clear dose-response relationship with osteomyelitis risk. This finding suggests that the observed association may not be fully accounted by limited physical activity, and may also be related to the coexistence of multiple unfavorable host-related factors. A multicenter matched study found that patients with osteomyelitis were more likely to suffer from malnutrition, hypoalbuminemia, and heavier chronic disease burdens, which in turn were associated with more postoperative complications, prolonged hospitalization, and greater challenges in infection management [13]. Meanwhile, frailty often coexists with multiple chronic comorbidities such as diabetes, cardiovascular disease, and chronic kidney disease; these conditions may not only aggravate systemic inflammatory responses and delay tissue repair, but also further weaken the host’s capacity to buffer and recover from infection [2]. Blyth et al. reported in a long-term epidemiological study that older adults with both frailty and chronic diseases exhibited a significantly higher incidence of osteomyelitis compared to the general population. Taken together, frailty may reflect not a single abnormality, but rather a vulnerable host state associated with the accumulation of comorbidities, nutritional imbalance, and internal homeostatic dysregulation; these factors may coexist and potentially contribute to a biological background linked to infection onset and persistence. Similarly, studies on chronic nonbacterial osteomyelitis (CNO), a type of autoinflammatory bone disorder, have identified sustained activation of pro-inflammatory cytokines (IL-1, TNF-α) as a cause of chronic inflammatory damage in bone tissue [21]. This persistent pro-inflammatory state is also relatively common among frail individuals. Therefore, the inflammation-related microenvironment associated with frailty may not be a static accompanying phenomenon, but may instead participate in an amplifying process in which persistent inflammation, progressive tissue injury, and impaired repair reinforce one another. This may provide another possible explanation for the association observed in our study.

From a clinical practice perspective, these findings suggest that frailty assessment may have potential value in helping to identify individuals at higher risk of osteomyelitis. Osteomyelitis causes severe complications and poor prognoses, such as prolonged hospitalization and significantly increased medical costs, which greatly increase treatment difficulty and healthcare resource consumption [22]. Notably, a recent clinical study using the modified frailty index (mFI-5) further supported the value of frailty status in infection risk assessment: the mFI-5 score was significantly associated with implant-related infection, prolonged hospitalization, and postoperative mortality [23]. The five frailty criteria used in our study are highly consistent with the mFI-5 structure, further suggesting the potential practical value of frailty assessment in infection risk stratification. Routine measurement of gait speed and grip strength, combined with brief frailty screening tools, may help early identify high-risk individuals and inform perioperative management [24,25]. For patients screening positive, further systematic assessment and multimodal interventions may be considered, particularly during perioperative and hospitalization periods, emphasizing exercise training combined with nutritional optimization, smoking cessation, glycemic control, and weight management. Randomized controlled trials and systematic reviews have demonstrated that such multimodal interventions in surgical patients significantly reduce postoperative complications and improve functional outcomes. Among these strategies, preoperative smoking cessation is especially important, effectively decreasing wound complications and infection risk [26–28]. In summary, systematic identification and targeted intervention for modifiable frailty-related risk factors may help inform perioperative care in individuals at higher risk of osteomyelitis and may potentially contribute to improved infection-related outcomes and long-term prognosis.

The major strengths of the present study include its large sample size and the consistent results in several sensitivity and subgroup analyses. This study also has some limitations. First, osteomyelitis diagnoses relied solely on electronic health records and ICD-10 codes from hospitalization data, potentially causing misclassification or omission of milder outpatient cases. In addition, the available data did not provide detailed information on the types or causes of osteomyelitis, such as hematogenous, post-traumatic, or diabetic foot-related infections, nor did they allow assessment of anatomical distribution, disease severity, or microbiological context. Therefore, the clinical interpretation of our findings is limited. Second, due to the observational design, residual confounding by unmeasured factors, such as inflammatory biomarkers, microbiological data, or disease severity, cannot be completely ruled out. Third, the UK Biobank participants are relatively healthy volunteers, potentially limiting generalizability to broader or clinical populations. Therefore, our findings need to be further validated in future prospective studies.

## Conclusion

In summary, results from this study indicate that both physical pre-frailty and frailty are associated with higher odds of osteomyelitis compared with physical non-frailty. A graded association was observed across frailty categories.

## Supporting information

S1 Table Physical frailty index criteria. (DOCX)

S2 Table ICD 10 codes for Osteomyelitis and other clinical risk factors (UK Biobank field ID: 41270). (DOCX)

S3 Table The numbers and percentages of participants with missing covariates. (DOCX)

S4 Table Odds ratios and 95% confidence intervals obtained from Models 1–4 for the association between physical frailty status and risk of osteomyelitis after excluding participants who developed osteomyelitis within the first two years of follow-up. (DOCX)

S5 Table Odds ratios and 95% confidence intervals obtained from Models 1–4 for the association between physical frailty status and risk of osteomyelitis after excluding individuals with missing covariate data. (DOCX)

S6 Table Odds ratios and 95% confidence intervals obtained from Models 1–4 for the association between physical frailty status and risk of osteomyelitis after performing multiple imputation for missing covariates. (DOCX)
