# Supplementary material for: Association between frailty status and osteomyelitis: A nested case-control study
Source: PLoS One. 2026 Jun 1;21(6):e0350395. doi: 10.1371/journal.pone.0350395 (PMC13225637; doi:10.1371/journal.pone.0350395)
Supplement: S1 Table — (DOCX) [file pone.0350395.s001.docx]

**Supplementary Table S1.** Physical frailty index criteria.

| **Physical frailty indicators** | **UK Biobank field ID** | **ACE touchscreen question** | **Physical frailty index** |
| --- | --- | --- | --- |
| Weight loss | 2306 | Compared with one year ago, has your weight changed? | - Lost weight=1 - Other=0 |
| Exhaustion | 2080 | Over the past two weeks, how often have you felt tired or had little energy? | - More than half the days or nearly every day=1 - Other=0 |
| Physical activity | 6164 | In the last 4 weeks did you spend any time doing the following? (You can select more than one answer) | - No activity=1 - Medium or heavy activity=0 |
|  | 1011 | How many times in the last 4 weeks did you do light do-it-yourself (DIY) (e.g. pruning, watering the lawn)? | - Light activity with a frequency of once per week or less=1 - Light activity more than once per week=0 |
| Walking pace | 924 | How would you describe your usual walking pace? | - Slow=1 - Other=0 |
| Grip strength | 31 | Sex of participant. | - Maximal grip strength of left and right hands. - Sex and body-mass index adjusted. |
|  | 46 | Left grip strength. |  |
|  | 47 | Right grip strength. |  |
|  | 21001 | BMI value here is constructed from height and weight measured during the initial Assessment Centre visit. |  |
