# Supplementary material for: Association between frailty status and osteomyelitis: A nested case-control study
Source: PLoS One. 2026 Jun 1;21(6):e0350395. doi: 10.1371/journal.pone.0350395 (PMC13225637; doi:10.1371/journal.pone.0350395)
Supplement: S2 Table — (DOCX) [file pone.0350395.s002.docx]

**Supplementary Table S2.** ICD 10 codes for Osteomyelitis and other clinical risk factors (UK Biobank field ID: 41270).

| **Outcome** | **Codes** |
| --- | --- |
| Osteomyelitis (total) | M462, M4620, M4621, M4622, M4623, M4624, M4625, M4626, M4627, M4628, M4629, M86, M860, M8600, M8601, M8602, M8603, M8604, M8605, M8606, M8607, M8608, M8609, M861, M8610, M8611, M8612, M8613, M8614, M8615, M8616, M8617, M8618, M8619, M862, M8620, M8621, M8622, M8623, M8624, M8625, M8626, M8627, M8628, M8629, M863, M8630, M8631, M8632, M8633, M8634, M8635, M8636, M8637, M8638, M8639, M864, M8640, M8641, M8642, M8643, M8644, M8645, M8646, M8647, M8648, M8649, M865, M8650, M8651, M8652, M8653, M8654, M8655, M8656, M8657, M8658, M8659, M866, M8660, M8661, M8662, M8663, M8664, M8665, M8666, M8667, M8668, M8669, M868, M8680, M8681, M8682, M8683, M8684, M8685, M8686, M8687, M8688, M8689, M869, M8690, M8691, M8692, M8693, M8694, M8695, M8696, M8697, M8698, M8699 |
| Diabetes mellitus | E10, E100, E101, E102, E103, E104, E105, E106, E107, E108, E109, E11, E110, E111, E112, E113, E114, E115, E116, E117, E118, E119, E12, E120, E121, E122, E123, E124, E125, E126, E127, E128, E129, E13, E130, E131, E132, E133, E134, E135, E136, E137, E138, E139, E14, E140, E141, E142, E143, E144, E145, E146, E147, E148, E149 |
| Chronic kidney disease | N18, N181, N182, N183, N184, N185, N189 |
| Immunosuppression | J45, J46, M05, M06, M08, K51, E10, I00-I02, I05-I09, L40, K900, K50, M353, G35, J301-J304, M790, M07, E050, E063, M45, M31, D86, L43, M350, L93, M32, D693, K743, M60, G610, G700, L10-L14, K754 |
| Multimorbidity | H90, H91, H810, M545, M255, G44, O294, O745, O894, R51, M543, M722, G560, M797, B02, M995, M4910, M4920, M45X9, M100, M101, M102, M103, M4712, M4782, M4792, G500, M50, M51, M510, M511, M5116, M5119, M512, M513, M5137, E78, I10, I11, I110, I119, I12, I120, I129, I13, I130, I131, I132, I139, I15, I150, I151, I152, I158, I159, I72, I73, G632, G590, H360, H280, E10, E11, E12, E13, E14, E00, J45, J450, J451, J458, J459, J41, J42, J43, J44, J47, F32, F33, F341, F381, F204, F40, F41, F43, F431, F42, Z733, G470, F99, F102, K70, F101, F111, F100, F112, F119, F20, F21, F30, G20, G21, G22, G23, G259, G26, G903, G35, G40, G43, G933, S00, S01, S02, S03, S04, S05, S06, S07, S08, S09, S020, S021, S028, S0291, S0402, S0403, S0404, S06, S071, T744, A80-A89, G00, G01, G02, G03, G04, G05, G06, G07, G08, G09, G37, I671, G809, I62, F50, F500, F501, F502, F503, F504, F505, F508, F509, K21, K210, K219, K20, K227, K228, K229, K23, K25, K29, K26, K30, Q401, B980, K57, D51, K590, E05, E03, E04, E01, E06, E07, E02, M60, G729, M32, D21, M35, M994, M997, M350, M33, L940, L941, M053, M058, M059, M06, M07, M353, M813, K900, M15, M150, M1500, M151, M16, M17, S327, S3270, S72, S820, S8200, K50, K51, K58, Q611, Q612, Q613, N17, N18, N19, E112, N028, N40, N41, N42, N510, F31, L20, L21, L22, L23, L24, L25, L26, L27, L30, L40, L41, J32, B16, B180, B181, B182, B172, B188, B189, B19, I85, B581, K701, K712, K713, K714, K715, K716, K74, K743, N80, E282, B20, B21, B22, B23, B24, H40, H25, H26, H28, H353 |
| Sickle cell disease | D57, D570, D571, D572, D573, D578 |
