# Supplementary material for: Association between frailty status and osteomyelitis: A nested case-control study
Source: PLoS One. 2026 Jun 1;21(6):e0350395. doi: 10.1371/journal.pone.0350395 (PMC13225637; doi:10.1371/journal.pone.0350395)
Supplement: S3 Table — (DOCX) [file pone.0350395.s003.docx]

**Supplementary Table S3.** The numbers and percentages of participants with missing covariates.

| **Variable** | **N** | **%** |
| --- | --- | --- |
| Education level | 71 | 1.11 |
| Smoking status | 17 | 0.27 |
| Alcohol intake | 7 | 0.11 |
| Healthy diet score | 257 | 4.01 |
| Vitamin D | 33 | 0.51 |
| Calcium supplementation | 16 | 0.25 |
