# Supplementary material for: Association between frailty status and osteomyelitis: A nested case-control study
Source: PLoS One. 2026 Jun 1;21(6):e0350395. doi: 10.1371/journal.pone.0350395 (PMC13225637; doi:10.1371/journal.pone.0350395)
Supplement: S4 Table — (DOCX) [file pone.0350395.s004.docx]

**Supplementary Table S4.** Odds ratios and 95% confidence intervals obtained from Models 1–4 for the association between physical frailty status and risk of osteomyelitis after excluding participants who developed osteomyelitis within the first two years of follow-up.

|  | **Case/Control** | **OR (95% CI)** | | | |
| --- | --- | --- | --- | --- | --- |
|  |  | **Model 1**ᵃ | **Model 2**ᵇ | **Model 3**ᶜ | **Model 4** ^d^ |
| **Non-frailty** | 354/3142 | 1.00 (Reference) | 1.00 (Reference) | 1.00 (Reference) | 1.00 (Reference) |
| **Pre-frailty** | 491/2029 | 2.22 (1.91-2.59) ^*^ | 1.91 (1.63-2.24) ^*^ | 1.90 (1.62-2.23) ^*^ | 1.38 (1.16-1.64) ^*^ |
| **Frailty** | 148/169 | 7.92 (6.13-10.22) ^*^ | 5.77 (4.37-7.62) ^*^ | 5.68 (4.30-7.50) ^*^ | 2.79 (2.05-3.81) ^*^ |
| ***P*-trend** |  | <0.001 | <0.001 | <0.001 | <0.001 |

ᵃ Adjusted for age (years) and sex (male or female).

ᵇ Basic model + ethnic background, Townsend Deprivation Index, education level, body mass index, smoking status, alcohol intake, healthy diet score.

ᶜ Model 2 + vitamin D supplementation, calcium supplementation.

^d^ Model 3 + diabetes mellitus, chronic kidney disease, immunosuppression, history of trauma or surgery, multimorbidity, and sickle cell disease

^*^ *P*-value < 0.001

CI, confidence interval; OR, odds ratio.
