# Supplementary material for: Association between frailty status and osteomyelitis: A nested case-control study
Source: PLoS One. 2026 Jun 1;21(6):e0350395. doi: 10.1371/journal.pone.0350395 (PMC13225637; doi:10.1371/journal.pone.0350395)
Supplement: S6 Table — (DOCX) [file pone.0350395.s006.docx]

**Supplementary Table S6.** *Odds ratios and 95% confidence intervals obtained from Models 1–4* for the association between physical frailty status and risk of osteomyelitis after performing multiple imputation for missing covariates.

|  | **Case/Control** | **OR (95% CI)** | | | |
| --- | --- | --- | --- | --- | --- |
|  |  | **Model 1**ᵃ | **Model 2**ᵇ | **Model 3**ᶜ | **Model 4** ^d^ |
| **Non-frailty** | 379/3142 | 1.00 (Reference) | 1.00 (Reference) | 1.00 (Reference) | 1.00 (Reference) |
| **Pre-frailty** | 531/2029 | 2.23 (1.93-2.58) ^*^ | 1.92 (1.64-2.23) ^*^ | 1.91 (1.64-2.23) ^*^ | 1.38 (1.16-1.64) ^*^ |
| **Frailty** | 158/169 | 7.98 (6.23-10.23) ^*^ | 5.83 (4.46-7.62) ^*^ | 5.80 (4.43-7.58) ^*^ | 2.78 (2.04-3.78) ^*^ |
| ***P*-trend** |  | <0.001 | <0.001 | <0.001 | <0.001 |

ᵃ Adjusted for age (years) and sex (male or female).

ᵇ Basic model + ethnic background, Townsend Deprivation Index, education level, body mass index, smoking status, alcohol intake, healthy diet score.

ᶜ Model 2 + vitamin D supplementation, calcium supplementation.

^d^ Model 3 + diabetes mellitus, chronic kidney disease, immunosuppression, history of trauma or surgery, multimorbidity, and sickle cell disease

^*^ *P*-value < 0.001

CI, confidence interval; OR, odds ratio.
